# Supplementary material for: Time series analysis of daily reported number of new positive cases of COVID-19 in Japan from January 2020 to February 2023
Source: PLoS One. 2023 Sep 15;18(9):e0285237. doi: 10.1371/journal.pone.0285237 (PMC10503708; doi:10.1371/journal.pone.0285237)
Supplement: Attachment — Submitted filename: Response_08_Aug_2023.docx [file pone.0285237.s002.docx]

Responses to reviewer comments

In response to the comments, the following text has been amended in the revised manuscript. These revisions are highlighted using bold type. The revised manuscript has been edited by professional editors at Edanz Group Ltd.

Reviewer #1

Comments:

- Why is the MEM-PSD method not limited by the sampling frequency in the data? A brief explanation would help the reader.
**Response: I apologize for the lack of clarity here that may have misled the reader into thinking that “the MEM-PSD method [is] not limited by the sampling frequency in the data.” In fact, the MEM spectral analysis method is limited by the sampling interval of the data and the MEM-PSD is calculated up to the Nyquist frequency. In the revised manuscript, I have added related text (page 6, lines 93-95) as follows:**

**“The MEM-PSD is calculated up to the Nyquist frequency (=1/(2Δ*t*)), corresponding to *f* = 182.5 (1/year) in the present study.”**

**Additionally, I have clarified the frequency range of the PSDs shown in Fig 2 and 4 in the revised manuscript as follows:**

**(page 11, line185): “... of the PSD ( *f* ≤ 50.0) are shown in Fig 2a and 2b…”**

**(page 13, line 216): “… of the PSDs ( *f* ≤ 6.0) in Fig 2a and 2b are …”**

- Fig 1, and page 9 line 145: The histogram in 1e does not look Gaussian, although it certainly looks better than the original data. Please discuss what limitations could the study have (or how the results could be impacted) by the non-Gaussianity.
**Response: I have examined whether the results obtained were impacted by non-Gaussianity by confirming the validity of the MEM spectral analysis results, and found that they were not impacted. I confirmed this by calculating the least squares fitting (LSF) curve pertaining to the original time series data *x*(*t*) with MEM-estimated periods. To indicate the procedure used for this confirmation, in the revised manuscript, I have added Fig 3, Table 1 (Pages 13-14), and a new subsection, “Dominant spectral lines,” in the “Results” section (page 12, lines 204-21) as follows:**

**“Dominant spectral lines**

**In the PSDs for phases I and II (Fig 2a and 2b), many well-defined spectral lines are clearly observed. Three dominant spectral-peak frequency modes for each phase are shown in Table 1, with corresponding periods and intensities (powers) of the peaks. For each phase, an LSF curve (Equation (3)) to the residual data (Fig 1d) was calculated with three dominant periods listed in Table 1. The LSF curves thus obtained for phases I and II were indicated in Fig 3a and 3b, respectively. In each figure, the LSF curve reproduces the residual data well. Thus, the periods detected by MEM spectral analysis for each phase (Fig 2a and 2b, Table 1) were confirmed to be accurate. The good fit of each LSF curve to the residual data (Fig 3a and 3b) was supported by the fact that the ρ values between the residual data and the LSF curve were large. The values of ρ were 0.86 and 0.79 for phases I and II, respectively.”**

**Table 1. Frequencies and powers of the three dominant spectral peaks shown in Fig 2a and 2b:** **(a) Phase I (pre-Tokyo Olympic Games, 11 February 2020–22 July 2021). (b) Phase II (post- Tokyo Olympic Games, 23 July 2021–21 February 2023).**

| **Variable** | ***f* (1/year)** | **Power** |
| --- | --- | --- |
| **Phase I** | **0.6**  **2.9**  **3.8** | **0.83**  **0.51**  **0.16** |
| **Phase II** | **0.6**  **1.6**  **2.2** | **0.87**  **0.80**  **1.13** |

**Regarding the result mentioned above, I have added new text in the “Discussion” section of the revised manuscript (page 19, lines 331-345). At the end of this new text, I have specified that the results obtained were not impacted by non-Gaussianity (Fig 1d’), which is noted as “The conversion of analyzed time series data to normal distribution (Fig 1d’) also contributes to precise determination of the MEM-estimated periods (Fig 3a and 3b, Table 1)”:**

**“****The validity of the periodic modes detected by MEM spectral analysis (Table 1) was confirmed by calculating the LSF curve to *x*(*t*) (Fig 3), which** **was expressed in the form of a linear combination of cosine functions in Equation (3). This curve is obtained from the Fourier transformation of the MEM-PSD, as described in Reference 16; that is, the following expression is obtained for the time series data:**

$x(t)=\sum_{j=1}^{N} \cos\left\{2\pi f_{j}\left(t+\theta_{j}\right)\right\}+\varepsilon(t),$ **(5)**

**where *f _j_* is the center frequency of the *j*-th peak intensity in MEM-PSD. The first term is identical to Equation (3), while the second term is undeterministic noise due to aperiodic or periodic fluctuations that cannot be expressed in the first term. This is the reason that the validity of the MEM spectral analysis results was confirmed by calculating the LSF curve with Equation (3) to *x*(*t*) with MEM-estimated periods. In this study, the good fit of the LSF curves with Equation (3) to the residual data (Fig 3a and 3b) indicates that the periodic structure of the residual data (Table 1) was determined precisely. The conversion of analyzed time series data to normal distribution (Fig 1d’) also contributes to precise determination of the MEM-estimated periods (Fig 3a and 3b, Table 1).****”**

- "Results" section: MEM-PSD is applied several times, either to data or to residuals. A more detailed description of the approach, in order that is applied should be provided, otherwise the reader is confused about where MEM is applied.
**Response: I have added a new subsection called “Outline of the analysis procedure” to the “Methods” section (page 9, lines 154-163):**

**“Outline of the analysis procedure**

**First, MEM spectral analysis was performed for the log-data (Fig 1b) and the long-term period was determined from the PSD for the log-data (Fig 1c). Then long-term trends in the log-data were calculated using the LSF method (Equation (3)) with the MEM-estimated period. This LSF curve (Fig 1b) that corresponded to the long-term trend was removed by subtracting the LSF curve from the log-data, and thus the residual data (Fig 1d) were obtained. Then the MEM-PSDs of phases I and II of the residual data were calculated (Fig 2a and 2b, respectively). Finally, segment time series analysis was performed and the MEM-PSD was obtained for each segment of the residual data (Fig 5 and 6).”**

- The preparation of the data could be moved to the methods part to make the results section clearer.
**Response: I have moved the subsection “Preparing the data for analysis” from the “Results” section to the “Methods” section (page 7, lines 112-134).**

- Page 10, line 165: how is lambda estimated?
**Response: I have added sentences to indicate how the *λ*-value was determined (page 11, lines 196-201) as follows:**

**“To obtain the magnitude of *λ*, the mean power of the PSD was calculated by integrating the PSD over small frequency interval *∆f*, that is, the mean power of the PSD is the power in the interval of frequencies [*f*, *f* +*∆f*]. The line of the PSD gradient is calculated as a regression line against the mean powers in the low frequency range (*f* ≤ 12.0), and is drawn in Fig 2a and 2b. The precise value of *λ* was determined using this procedure.”**

- Page 11, line 182: the method is more commonly now as "segmented time series analysis". The method should be presented in the methods section.
**Response: I have created a new subsection called “Segment time series analysis” in the “Methods” section and moved the following sentences from the “Results” section to the “Methods” section (page 9, lines 146-152).**

**“Segment time series analysis**

**We further investigated periodic structures of the residual data (Fig 1d) with segment time series analysis. The residual data (Fig 1d) were divided into several segments and the MEM-PSD for each segment was computed. In this study, each segment represents a 365-day time interval, with the starting point of two consecutive segments delayed by 6 days. The MEM-PSDs thus obtained were arranged in the order of the time sequence to construct a 3-dimensional (3D) spectral array.**”

- Page 14, line 239: rather than "is based", one would say "is dominated" or similar; real data is certainly not based on a model!
**Response: I have revised this sentence (page 17, lines 294-295) as follows:**

**“**… in Japan is **dominated** **by** deterministic nonlinear dynamics.”

- Page 15, line 255: Using "study" twice is confusing. Change to "A detailed study investigating chaotic characteristics for each prefecture in Japan is in preparation", or similar.
**Response: I have revised this sentence (page 18, line 311) as follows:**

“… prefecture in Japan is **in the preparation phase**.”

- Page 16, line 270: characteristic (rather than peculiar).
**Response: I have revised this sentence (page 21, line 359) as follows:**

“…, which **is characteristic of** the nonlinear … .”

Page 2, line 19 (and elsewhere): variational typically refers to optimization for functionals; here a more common expression would be "variation" or "variability", or even just "temporal structures".
**Response: I have changed “temporal variational structure” to “temporal variation” in the revised manuscript as follows:**

**Page 2, lines 11, 19**

**Page 3, lines 42, 45, 49**

**Page 4, line 52**

**Page 10, lines 176-177**

**Page 16, line 280**

**Page 21, line 354**

Page 2, line 27: The sentence about measles control needs a little bit of context.
**Response: I have revised this sentence (page 2, lines 27-28) as follows:**

“**Additionally, the** **possibility of applying theoretical studies for measles control programs to COVID-19 is discussed.**”

Page 3, line 34: "recurrently" (not recursively)
**Response: I have revised this sentence (page 3, line 34) as follows:**

“… also known as COVID-19, still occur **recurrently**, … .”

Page 4, line 61: Could you mention the total population (or what percentage of the total population the total number of cases represents)?

**Response: I have added a new sentence (page 4, lines 60-62) and corrected the total from 32,851,734 to 33,119,744 as follows:**

“During this period, a total **33,119,744** new positive cases of COVID-19 were reported in Japan**, which represents 26.5% of the country’s total population of 125 million.**”

Reviewer #2:

(a) The main concern: There have been extensive studies on time series analysis for daily COVID-19 data for almost every region with different methodologies. The manuscript fails to present more details to compare the methodology in the current with other approaches, as well as the results of the current study and other existing ones for Japanese pandemic pattern.
    **Response: To compare the methodology and result in the current with other approaches, I have added a new sentence in the “Discussion” section (page 18, lines 312-322) and seven new references (reference numbers 34-40) as follows:**

**“****To investigate the temporal variations of the COVID-19 pandemic, studies have been conducted using interrupted time series analysis [34, 35] and the autoregressive integrated moving average (ARIMA) model [36]. Another important approach of time series analysis is the Bayesian spectral estimation method [37]. By contrast, in other studies, researchers interpreted the temporal variations of the COVID-19 pandemic using the SEIR model [38, 39], which is a well-known nonlinear dynamical system for epidemics of infectious diseases [40]. However, the interrupted time series analysis and ARIMA model using random noise, and the Bayesian spectral estimation method assuming approximate linearity of the time series data of COVID-19 cases, have a weakness in the interpretation of multiple periodicities of the time series data (Table 1) with characteristic fluctuations caused by nonlinear dynamics.”**

**(New references):**

34. **Vokó Z, Pitter JG. The effect of social distance measures on COVID-19 epidemics in Europe: an interrupted time series analysis. GeroScience 2020; 42: 1075-7082.**
35. **Islam N, Sharp SJ, Chowell G, Shabnam S, Kawachi I, Lacey B, et al. Physical distancing interventions and incidence of coronavirus disease 2019: natural experiment in 149 countries. BMJ 2020; 370: m27431.**
36. **Chyon FA, Suman NH, Fahim RI, Ahmmed S. Time series analysis and predicting COVID-19 affected patients by ARIMA model using machine learning. J Virol Methods 2022; 301: 114433.**
37. **Nason G. COVID-19 cycles and rapidly evaluating lockdown strategies using spectral analysis. Sci Rep 2020; 10: 22134.**
38. **He S, Peng Y, Sun K. SEIR modeling of the COVID-19 and its dynamics. Nonlinear Dyn. 2020; 101: 1667-1680.**
39. **Wang A, Jamal SS, Yang B, Pham VT. Complex behavior of COVID-19’s mathematical model. Eur Phys J Spec Top 2022; 231: 885-891.**
40. **Sumi A, Ohtomo N, Tanaka Y. Study on chaotic characteristics of incidence data of measles. Jpn J Appl Phys 1997; 36: 7460-7472.**

**With respect to other approaches for investigating the Japanese pandemic pattern, I have added a new sentence in the “Discussion” section (page 19, lines 322-330), as follows:**

**“The method Sapkota et al. applied to the time-series data of COVID-19 in Japan [8], the 0-1 test, is useful for examining whether the time series data is chaotic; however, this method is not suitable for detecting the multiple periodicities of the time series data (Table 1). In contrast, the present method based on MEM spectral analysis made it possible to identify periodicities in short time series with a high degree of frequency resolution [16]. In particular, the results obtained from segment time series analysis in the present study (Fig 5 and 6) showed that the periodic structures of the COVID-19 pandemic experience is changing over time.”**

(b) The data in the supplementary material are daily incidence, however, it was mentioned in L82 that "monthly time series data". Overall, different time units are used, including day (daily date), month and year (for frequency and period).
**Response:** I **apologize for this mistake. I have corrected this in the revised manuscript (page 5, line 83) as follows:**

“To investigate temporal patterns of *x*(*t*) in the **daily** time series data,”

**In the revised manuscript, I only used the unit “year” in the “Results” section, except for sentences that explain the meaning of the obtained result (page 13, lines 220-225) for the time unit, and corrected the values of frequencies *f*=2.8 and 2.0 to *f*=2.9 and 2.2, respectively, as follows:**

**“For phase I, the spectral peak at *f* =2.9 corresponds to the period 4.1-month, which reflects intervals of four large waves of the COVID-19 pandemic with peaks during April 2020 – May 2021 observed in the inset of Fig 1a. For phase II, the spectral peak at *f* =2.2 corresponds to the period 5.4-month, which reflects intervals of four large waves of the COVID-19 pandemic with peaks during August 2021 – January 2023 observed in Fig 1a.”**

(b) formula in equation (4) of L165 is not appropriately shown

**Response: I apologize for this mistake. I have corrected this in the revised manuscript (page 11, line 194) as follows:**

**“**	$P(f)\sim exp(-\lambda f)$	**(4)	”**

Reviewer #3

Concerning line 118, it appears that the date mentioned, June 30, 2023, may be a typographical error and should likely be corrected to 2021.
**Response: I apologize for this mistake. I have corrected this in the revised manuscript (page 8, line 138).**

It is suggested to consider presenting Figure 1b as an inset within Figure 1a, if feasible.
**Response: I have presented Fig 1b in the original manuscript as an inset within Fig 1a in the revised manuscript. Accordingly, I have changed the figure subscripts of Fig 1c, 1d, 1e, and 1e’ in the original manuscript to Fig 1b, 1c, 1d, and 1d’, respectively, in the revised manuscript. Accordingly, the figure caption of Fig 1a in the revised manuscript (page 8, lines 137-138) is as follows:**

“(a) **Original data, [inset] enlargement of the original data from 16 January 2020 to 30 June 2021**.”

In Figure 1e, it is recommended to include a vertical dashed line to delineate Phase I and Phase II. Additionally, annotating the phases would enhance clarity and understanding.
**Response: Please note that Fig 1e in the original manuscript is now labeled Fig 1d in the revised manuscript. In Fig 1d, I have included a vertical dashed line to delineate Phase I and Phase II, and added the labels of Phase I and Phase II (annotation).**

Regarding Figure 2a and 2b, annotating Phase I and Phase II directly within the figures would be helpful. Furthermore, the presence of two lines for low frequencies in both figures raises questions about their representation. It is advised to address this in the text by explaining whether they indicate a specific trend or any other relevant information.
**Response: I have added the labels of Phase I and Phase II (annotation) in Fig 2a and 2b in the revised manuscript. For the two lines in the low-frequency range in both figures, I have added sentences (page 11, lines 196-201) and revised the figure captions of Fig 2a and 2b (page 11 lines 185-189) as follows:**

**(page 11, lines 196-201):** “**To obtain the magnitude of *λ*, the mean power of the PSD was calculated by integrating the PSD over small frequency interval *∆f*, that is, the mean power of the PSD is the power in the interval of frequencies [*f*, *f* +*∆f*]. The line of the PSD gradient is calculated as a regression line against the mean powers in the low frequency range (*f* ≤ 12.0), and is drawn in Fig 2a and 2b. The precise value of *λ* was determined using this procedure.**”

**(page 11, lines 185-189)**: “**Fig 2.** **MEM-PSDs for two ranges of the residual data (*f* ≤ 50.0)**. (a) Phase I (pre-Tokyo Olympic Games, 11 February 2020–22 July 2021). (b) Phase II (post-Tokyo Olympic Games, 23 July 2021- 21 February 2023). **In each figure, the blue line in the low-frequency range (*f* ≤ 12.0) indicates the regression line with gradient *λ* of Equation (4). See the text for details.”**

For Figure 3a and 3b, where f = 2.8 and 2.0, it is suggested to provide explanations in the text regarding these values in the context of the original time series of daily COVID data. Specifically, clarifying whether the time series data represents new cases or includes repeat cases would be valuable information that seems to be missing from the text.
**Response: Please note that Fig 3 in the original manuscript is now labeled Fig 4 in the revised manuscript. I have added a sentence to explain the meaning of the spectral peaks (page 13, lines 220-225) and corrected the values of frequencies *f* = 2.8 and 2.0 to *f* = 2.9 and 2.2, respectively, as follows:**

**“****For phase I, the spectral peak at *f* =2.9 corresponds to the period 4.1-month, which reflects intervals of four large waves of the COVID-19 pandemic with peaks during April 2020 – May 2021 observed in the inset of Fig 1a. For phase II, the spectral peak at *f* =2.2 corresponds to the period 5.4-month, which reflects intervals of four large waves of the COVID-19 pandemic with peaks during August 2021 – January 2023 observed in Fig 1a.”**

**I have made it clear that the time series data represent the number of new cases as follows:**

**Page 1, Title “**Time series analysis of **daily reported number of new positive cases of COVID-19** in Japan from January 2020 to February 2023**”**

**Page 4, lines 58-59,**

**Page 5, line 75**

**Page 7, line 113**

**Page 8, line 136**

**Page 10, line 166-167**

**Page 18, line 294**

Enhancing the labels and axes in Figure 4 is recommended to improve readability and comprehensibility.
**Response: Please note that Fig 4 in the original manuscript is now labeled Fig 5 in the revised manuscript. In Fig 5 in the revised manuscript, I have indicated the value of the period (year) that corresponds to the value of the frequency (1/year) of the horizontal axis, and enhanced the labels on the right and left vertical axes.**

Notably, Figure 5 is considered the crux of the paper. To enhance its presentation, the addition of three horizontal dashed lines representing different means is proposed. Consistency can be maintained by using the blue color scheme used throughout the paper.
**Response: Please note that Fig 5 in the original manuscript is now labeled Fig 6 in the revised manuscript. I have added three horizontal dashed lines to Fig 6 in the revised manuscript. When I used blue for the three horizontal dashed lines, it was not immediately visible; hence, I have used red. Accordingly, I have revised the figure caption of Fig 6 (page 15, lines 256-258) as follows:**

**“****Fig 6. Temporal variations of the frequencies of dominant spectral peaks detected in the frequency range of 1.0 ≤ *f* ≤ 4.0. The three red dashed lines indicate *f* = 3.0 before May 2021, *f* = 2.2 from July to November 2021, and *f* = 2.0 thereafter.”**

Regarding the statement made in lines 95-97 about confirming the validity of MEM spectral analysis results through least squares fitting (LSF) curve calculation pertaining to the original time series data x(t) with MEM-estimated periods, it would be beneficial to support this claim by referencing other works. Alternatively, if other comparative methods or known signals are not available for this specific dataset, it would be appropriate to suggest alternative methods like comparing with results from other analysis techniques such as Fourier analysis (e.g., periodograms, Welch's method).
**Response: To support our claim, that is, “the validity of MEM spectral analysis results through least squares fitting (LSF) curve calculation pertaining to the original time series data x(t) with MEM-estimated periods”, in the revised manuscript, first, I have refereed the theory, as described in new paragraphs in the “Methods” section (page 7, lines 109-110) and “Discussion” section (page 19, lines 331-345), as follows:**

**(page 7, lines 109-110 for the “Methods”): “The expression of Equation (3) is derived from the Fourier transformation of the MEM-PSD as described in the “Discussion” section.**”

**(page 19, lines 331-345 for the “Discussion”):**

**“****The validity of the periodic modes detected by MEM spectral analysis (Table 1) was confirmed by calculating the LSF curve to *x*(*t*) (Fig 3), which** **was expressed in the form of a linear combination of cosine functions in Equation (3). This curve is obtained from the Fourier transformation of the MEM-PSD, as described in Reference 16; that is, the following expression is obtained for the time series data:**

$x(t)=\sum_{j=1}^{N} \cos\left\{2\pi f_{j}\left(t+\theta_{j}\right)\right\}+\varepsilon(t),$ **(5)**

**where *f _j_* is the center frequency of the *j*-th peak intensity in MEM-PSD. The first term is identical to Equation (3), while the second term is undeterministic noise due to aperiodic or periodic fluctuations that cannot be expressed in the first term. This is the reason that the validity of the MEM spectral analysis results was confirmed by calculating the LSF curve with Equation (3) to *x*(*t*) with MEM-estimated periods. In this study, the good fit of the LSF curves with Equation (3) to the residual data (Fig 3a and 3b) indicates that the periodic structure of the residual data (Table 1) was determined precisely. The conversion of analyzed time series data to normal distribution (Fig 1d’) also contributes to precise determination of the MEM-estimated periods (Fig 3a and 3b, Table 1).”**

**Next, I have applied this theory to the analyzed data in the revised manuscript. Therein, I have added new paragraph “Dominant spectral lines” in the “Results” section, Fig 3, and Table 1 to confirm the validity of the MEM spectral analysis results through a LSF curve calculation pertaining to the original time series data *x*(*t*) with MEM-estimated periods (page 7, lines 107-109 for the “Methods” section, page 12, lines 204-215 for the “Results,” and pages 13-14 for Table 1) as follows:**

**(Page 7, lines 107-109 for the “Methods”): “****The reproducibility level of *x*(*t*) from the optimum LSF curve was evaluated using Pearson’s correlation (ρ) with SPSS v. 17.0J software (SPSS, Japan).”**

**(Page 12, lines 204-205 for the “Results”):**

**“Dominant spectral lines**

**In the PSDs for phases I and II (Fig 2a and 2b), many well-defined spectral lines are clearly observed. Three dominant spectral-peak frequency modes for each phase are shown in Table 1, with corresponding periods and intensities (powers) of the peaks. For each phase, an LSF curve (Equation (3)) to the residual data (Fig 1d) was calculated with three dominant periods listed in Table 1. The LSF curves thus obtained for phases I and II were indicated in Fig 3a and 3b, respectively. In each figure, the LSF curve reproduces the residual data well. Thus, the periods detected by MEM spectral analysis for each phase (Fig 2a and 2b, Table 1) were confirmed to be accurate. The good fit of each LSF curve to the residual data (Fig 3a and 3b) was supported by the fact that the ρ values between the residual data and the LSF curve were large. The values of ρ were 0.86 and 0.79 for phases I and II, respectively.”**

**Table 1. Frequencies and powers of the three dominant spectral peaks shown in Fig 2a and 2b:** **(a) Phase I (pre-Tokyo Olympic Games, 11 February 2020–22 July 2021). (b) Phase II (post- Tokyo Olympic Games, 23 July 2021–21 February 2023).**

| **Variable** | ***f* (1/year)** | **Power** |
| --- | --- | --- |
| **Phase I** | **0.6**  **2.9**  **3.8** | **0.83**  **0.51**  **0.16** |
| **Phase II** | **0.6**  **1.6**  **2.2** | **0.87**  **0.80**  **1.13** |
